# Supplementary material for: Transgenic NADH dehydrogenase restores oxygen regulation of breathing in mitochondrial complex I-deficient mice
Source: Nat Commun. 2023 Mar 1;14:1172. doi: 10.1038/s41467-023-36894-2 (PMC9977773; doi:10.1038/s41467-023-36894-2)
Supplement: Supplementary file 1 — Supplementary Information [file 41467_2023_36894_MOESM1_ESM.pdf]

Ms. NCOMMS-22-20804B

Supplementary Information (Figures 1-11)

Transgenic NADH dehydrogenase restores oxygen regulation of breathing in mitochondrial complex I-deficient mice

Blanca Jiménez-Gómez[1,2,3, #], Patricia Ortega-Sáenz[1,2,3,#], Lin Gao[1,2,3], Patricia González-Rodríguez[1,2,3], Paula García-Flores[1,2,3], Navdeep Chandel[4] and José López-Barneo[1,2,3,*]

1. Instituto de Biomedicina de Sevilla (IBiS), Hospital Universitario Virgen del Rocío/CSIC/Universidad de Sevilla, 41013 Seville, Spain
2. Departamento de Fisiología Médica y Biofísica, Facultad de Medicina, Universidad de Sevilla, 41009 Seville, Spain
3. Centro de Investigación Biomédica en Red sobre Enfermedades Neurodegenerativas (CIBERNED), 28031 Madrid, Spain
4. Department of Pediatrics, Northwestern University, Chicago, IL 60611, USA

# These authors contributed equally

* Corresponding author

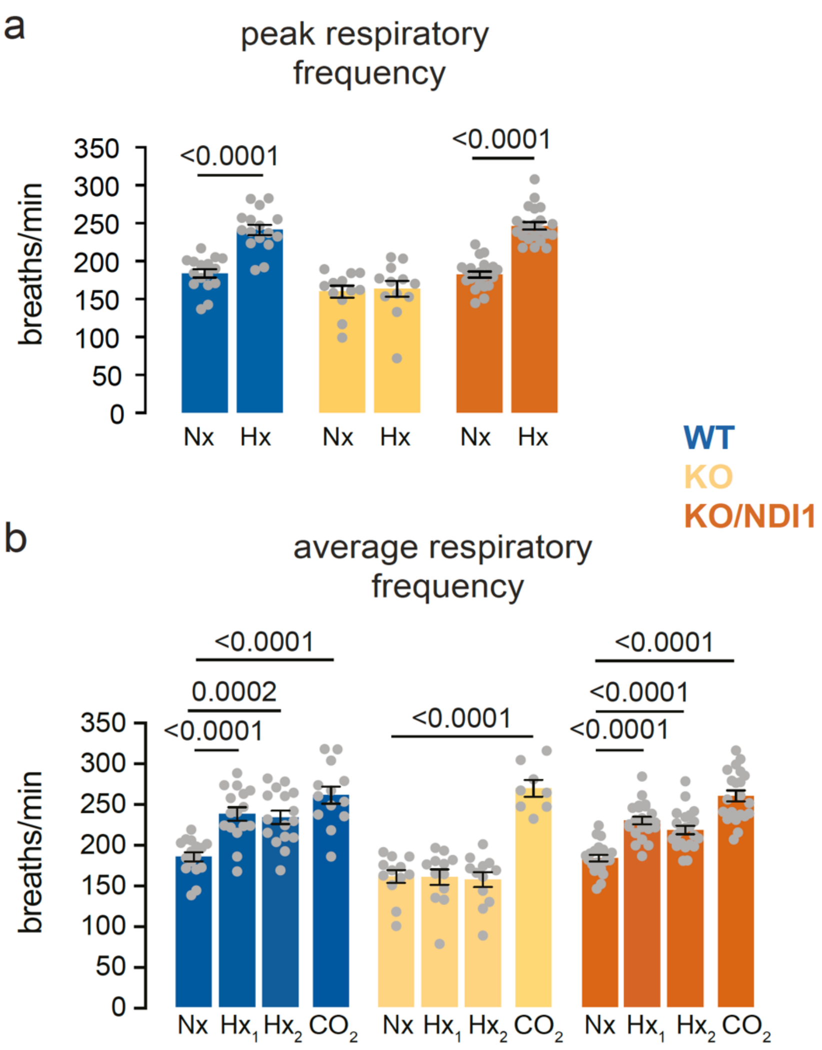

Supplementary Fig. 1. Quantitative measurements of acute ventilatory responses to hypoxia and hypercapnia in the mouse strains studied. a, Peak respiratory frequency (breaths/minute) recorded in normoxia (Nx, 21% O_2) and during hypoxia (Hx, 10% O_2) measured by plethysmography. Data points are represented as grey dots. Mean±SEM values are: WT mice (Nx: 184±6, n=16; Hx: 241±7, n=16), KO mice (Nx: 160±8, n=12; Hx: 164±10, n=12), KO/NDI1 mice (Nx: 182±4, n=22; Hx: 247±5, n=22). P-values calculated by two-tailed paired t test. **b,** Average respiratory frequency (breaths/minute) recorded in normoxia (Nx), during the first 80 s (Hx_1) or 300 s (Hx_2) after reaching 10% O_2 in the chamber, and during hypercapnia (CO_2) (see Methods). Mean±SEM values are (values in normoxia are the same as in panel **a**): WT mice (Hx_1: 236±8, n=16; Hx_2: 232±9, n=16; CO_2: 259±11, n=13), KO mice (Hx_1: 159±10, n=12; Hx_2: 156±9, n=12; CO_2: 268±12, n=8 mice), KO/NDI1 mice (Hx_1: 228±5, n=22; Hx_2: 217±5, n=22; CO_2 258±7, n=21). Data points are represented as grey dots. P values calculated by one-way ANOVA followed by Dunnett's multiple comparisons test are represented in each panel. Source data are provided in a Source Data file.

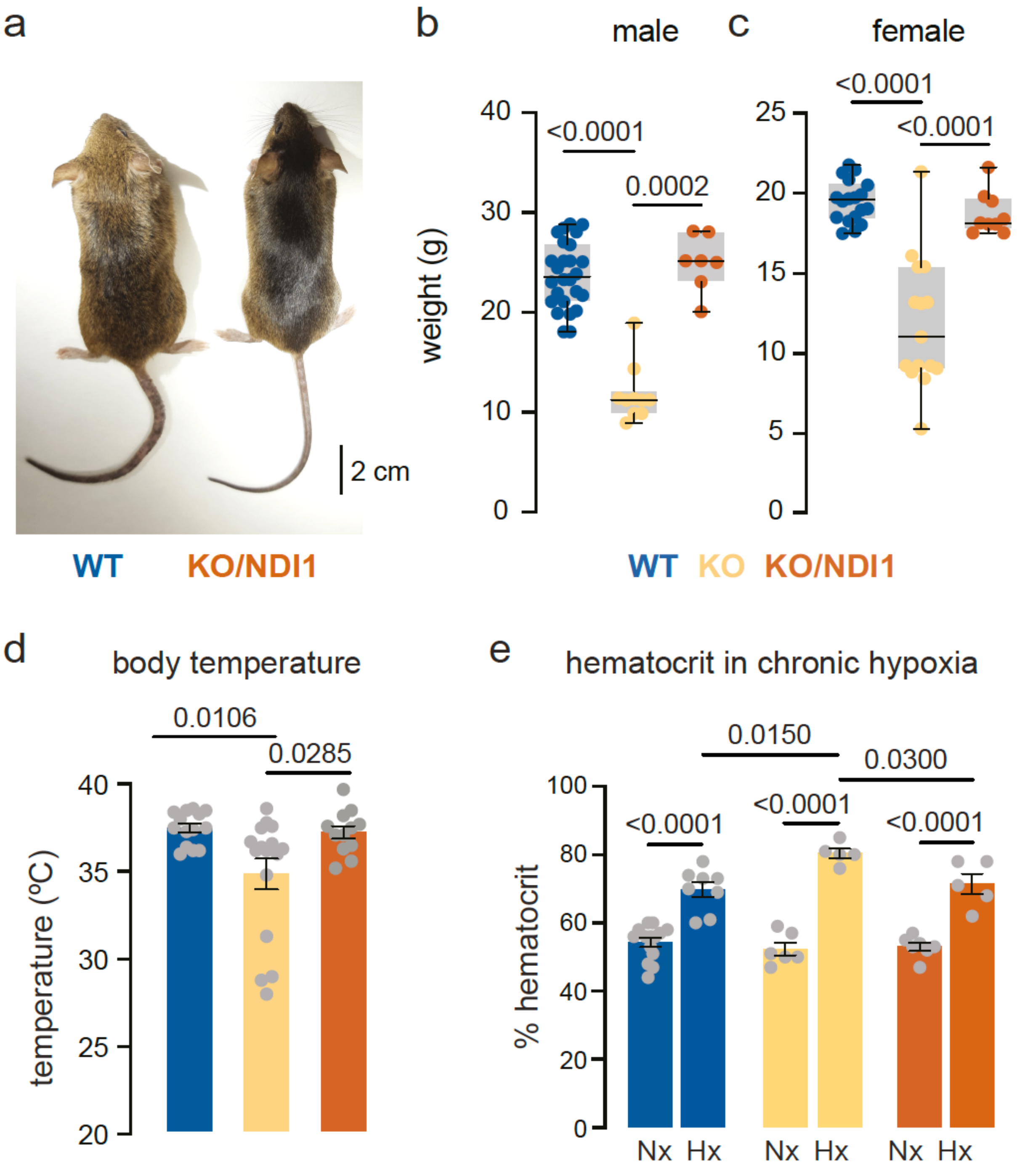

Supplementary Fig. 2. Recovery of systemic alterations in MCI-deficient mice by transgenic NDI1 expression. a, Representative images of WT (108 days old) and KO/NDI1 (115 days old) mice of similar age. All KO mice died before 75 days of age. **b, c,** Box plots representing distribution of weight, in grams (g), from male (**b**) and female (**c**) mice between 55 to 75 days of age. The plots

indicate median (middle line), 25th, 75th percentile (box), and largest and smallest values range (whiskers). All data points are superimposed. **b,** WT (n=26), KO (n=10), KO/NDI1 (n=7); **c,** WT (n=18), KO (n=15), KO/NDI1 (n=9). P values calculated with one-way ANOVA followed by Dunn's (male) or Tukey's (female) multiple comparisons tests are represented in each panel. **d,** Body temperature measurements (in Celsius degrees, ºC) in the three mouse models studied. Data points are represented as grey dots. Data are expressed as mean±SEM: (WT, blue, 37.3±0.2, n=14; KO, yellow, 34.7±0.9, n=16; KO/NDI1, brown, 37.1±0.4, n=12 mice). P-values calculated by one-way ANOVA followed by Tukey's multiple comparisons post hoc test. **e,** Hematocrit values obtained during normoxia (Nx) and after 10 days of chronic hypoxia (Hx, 10% O_2) in WT mice (blue, Nx: 54±1%, n=14; Hx: 70±2 %, n=8), KO mice (yellow, Nx: 52±2%, n=6; Hx: 80±1%, n=5) and KO/NDI1 mice (brown, Nx: 53±1%, n=7; Hx: 71±3%, n=5). Data are presented as mean±SEM with statistically significant P values (>0.05) superimposed. In **e**, comparison of values between Nx and Hx in each mouse model was done by two-tails unpaired t tests. Comparison of the values in Hx in the different mouse strains was done by one-way ANOVA followed by Newman-Keuls multiple comparisons post hoc test. Source data are provided in a Source Data file.

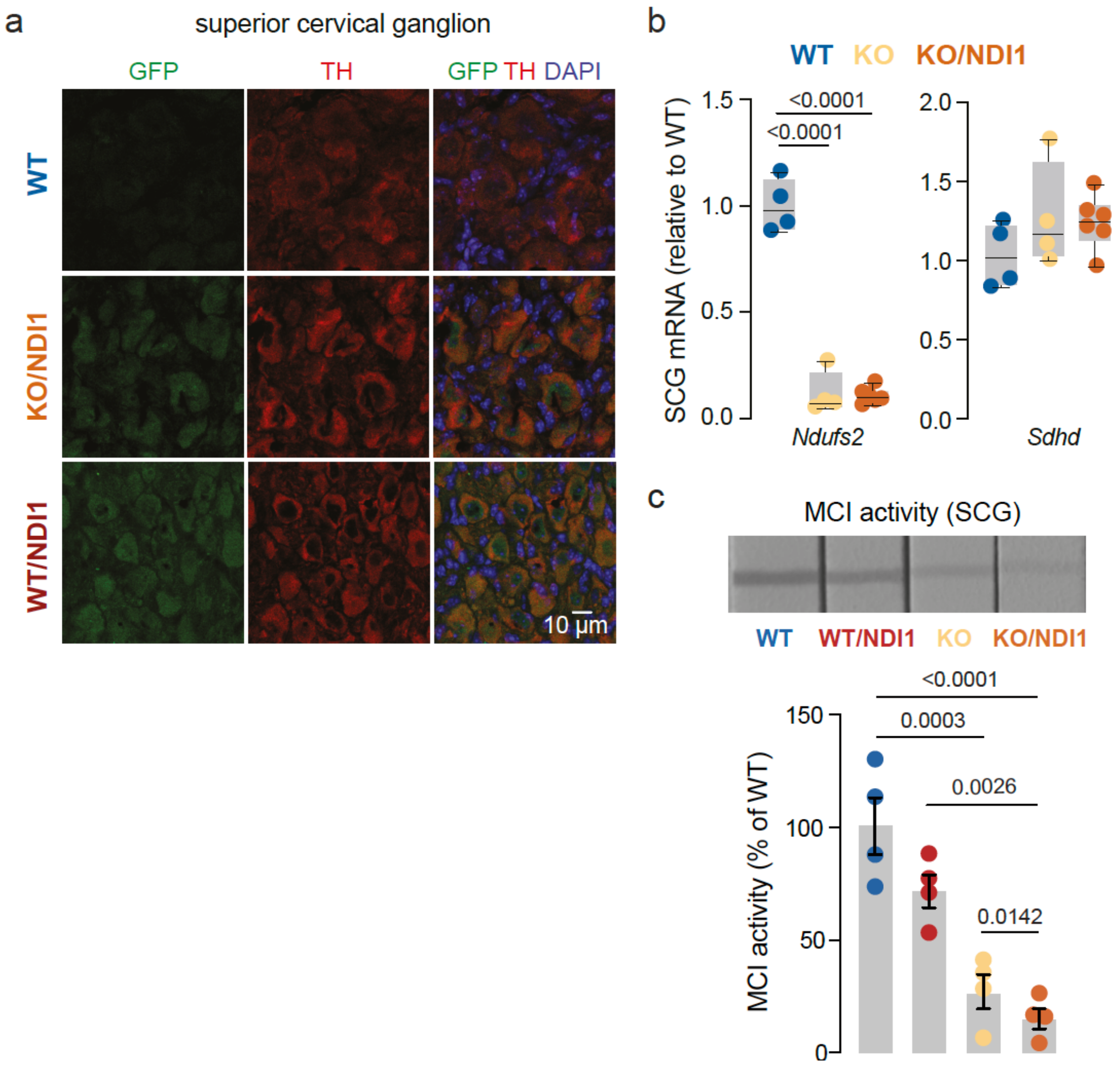

Supplementary Fig. 3. NDI1 expression in superior cervical ganglion neurons. a, Histological sections of the superior cervical ganglion (SCG) from WT (top), KO/NDI1 (middle) and WT/NDI1 (bottom) mice illustrating colocalization of NDI1 expression (green fluorescent protein, GFP) and tyrosine hydroxylase (TH). DAPI was used to stain nuclei. Similar immunocytochemical studies were performed in n=4 mice for each genotype. Calibration bar (10 μM) applies to all panels. **b,** Box plots representing *Ndufs2* (left) and subunit D of succinate dehydrogenase (*Sdhd)* (right) mRNA levels, relative to WT, in SCG samples from WT mice (blue dots, *Ndufs2*: n=4, *Sdhd*: n=4 replicates/group), KO mice (yellow dots, *Ndufs2*: n=4, *Sdhd*: n=4 replicates/group) and KO/NDI1 mice (brown dots, *Ndufs2*: n=6, *Sdhd*: n=6 replicates/group). The box plots indicate median (middle line), 25th, 75th percentile (box), and largest and smallest values range (whiskers). P-values were calculated by one-way ANOVA followed by Tukey's multiple comparisons post-hoc test. Mean±SEM values are: WT

mice (*Ndufs2*, 1±0.06; *Sdhd,* 1±0.1*),* KO mice (*Ndufs2,* 0.12±0.05; *Sdhd*, 1.24±0.17), KO/NDI1 mice (*Ndufs2,* 0.11±0.02; *Sdhd*, 1.21±0.07). **c,** Upper panel. Dipstick measurement of mitochondrial complex I (MCI) activity in SCG from the various mice strains studied. Lower panel. Quantification of MCI activity. Mean±SEM values (in % of WT) are: WT mice (100±13, n=4 replicates/group), KO mice (27±8, n=4 replicates/group), KO/NDI1 mice (15±4, n=4 replicates/group), WT/NDI1 mice (71±7, n=4 replicates/group). P-values, calculated by one-way ANOVA followed by Tukey's multiple comparisons post hoc test, are indicated. Source data are provided in a Source Data file.

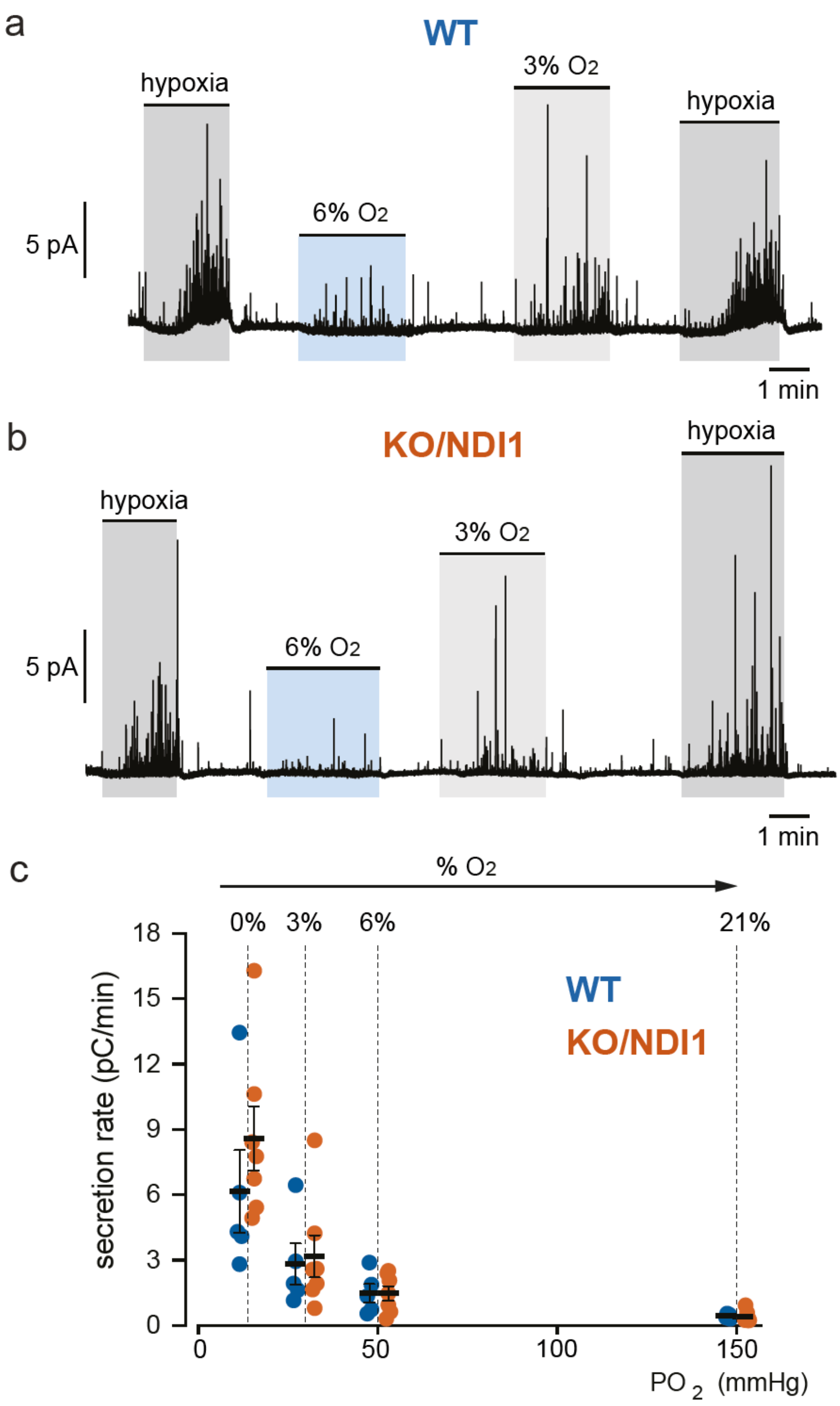

Supplementary Fig. 4. Responsiveness of CB glomus cells from WT and KO/NDI1 mice to different levels of O_2 tension. a, b, Representative amperometric recordings of dopamine secretion in glomus cells in CB slices from WT (**a**) and KO/NDI1 mice (**b**) bathed by solutions with variable levels of O_2 tension: Hypoxia (0% O_2, ~15 mmHg in the recording chamber), 3% O_2 (~30 mmHg in

the recording chamber), 6% O_2 (~50 mmHg in the recording chamber); Normoxia (21% O_2, 150 mmHg in the recording chamber) see Methods. **c,** Secretion rate at the different values of O_2 tension. Data, in picoCoulombs/minute, are expressed as mean±SEM with data values superimposed. 0% O_2 (WT, 5.9±1.9, n=5/4 cells/mice, KO/NDI1 8.4±1.5, n=7/4 cells/mice); 3% O_2 (WT, 2.6±0.9, n=5/4 cells/mice, KO/NDI1 3±1, n=6/4 cells/mice); 6% O_2 (WT, 1.3±0.4, n=5/4 cells/mice, KO/NDI1 1.3±0.3, n=7/4 cells/mice) 21%O_2 (WT, 0.24±0.04, n=5/4 cells/mice, KO/NDI1 0.21±0.1, n=7/4 cells/mice). Source data are provided in a Source Data file.

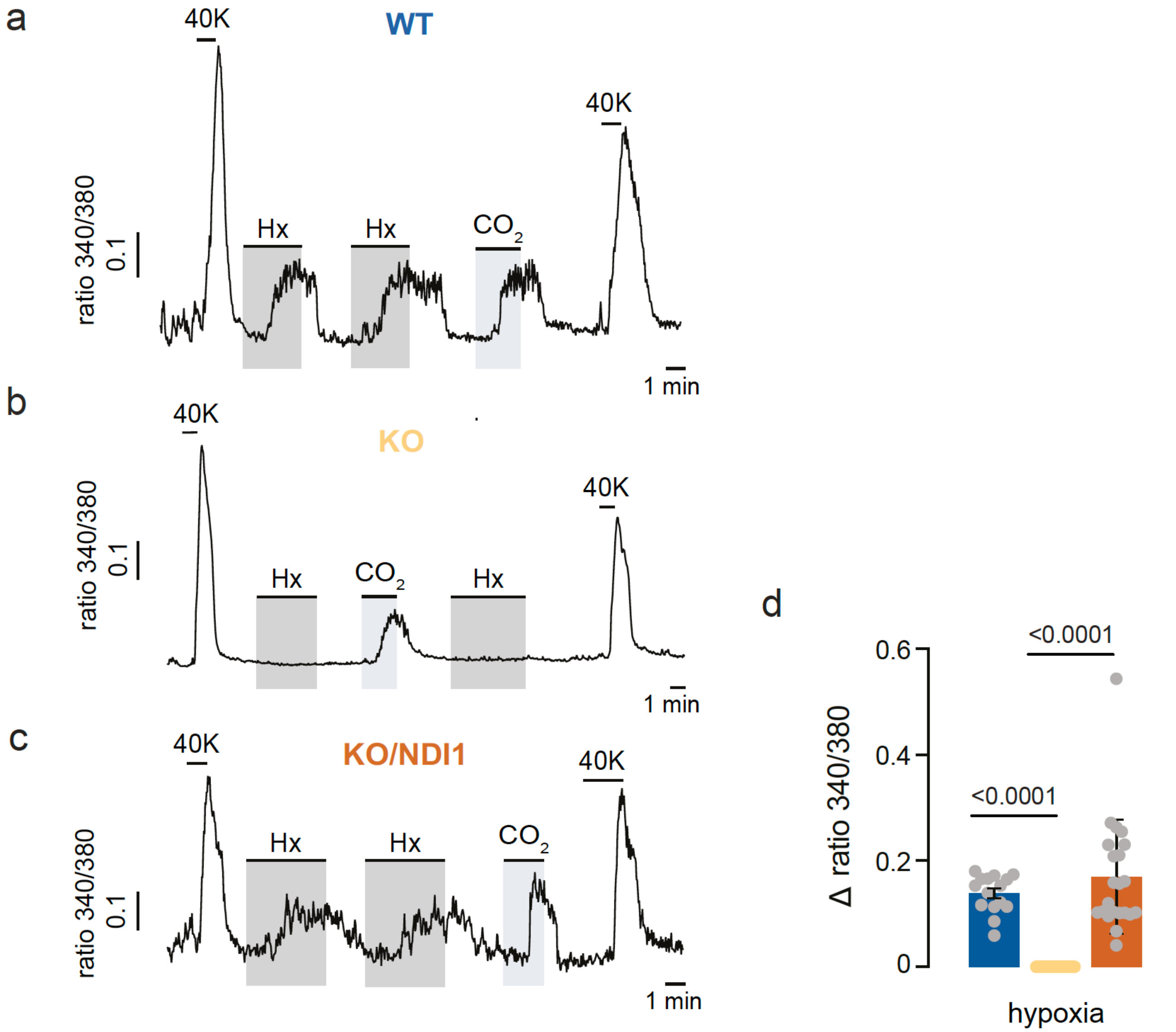

Supplementary Fig. 5. NDI1 expression restores the selective abolition of hypoxia-induced increase in cytosolic Ca^{2+} in complex I-deficient glomus cells. a, b, c, Representative recordings of the increases in cytosolic [Ca^{2+}] elicited by hypoxia (Hx), hypercapnia (CO_2) and high K^+ (40K) in Fura 2-loaded glomus cells from control (**a**), KO (**b**), and KO/NDI1 (**c**) mice. **d,** Quantification of the increase in cytosolic [Ca^{2+}] induced by hypoxia. Data are expressed as mean±SEM with all data points superimposed (grey dots). WT mice (blue, 0.14±0.009, n=15/3 cells/mice), KO mice (yellow, 0±0, n=31/3 cells/mice) and KO/NDI1 mice (brown 0.17±0.023, n=22/4 cells/mice). Indicated P values were calculated with Kruskal-Wallis test followed by Dunn's multiple comparisons post hoc test. Source data are provided in a Source Data file.

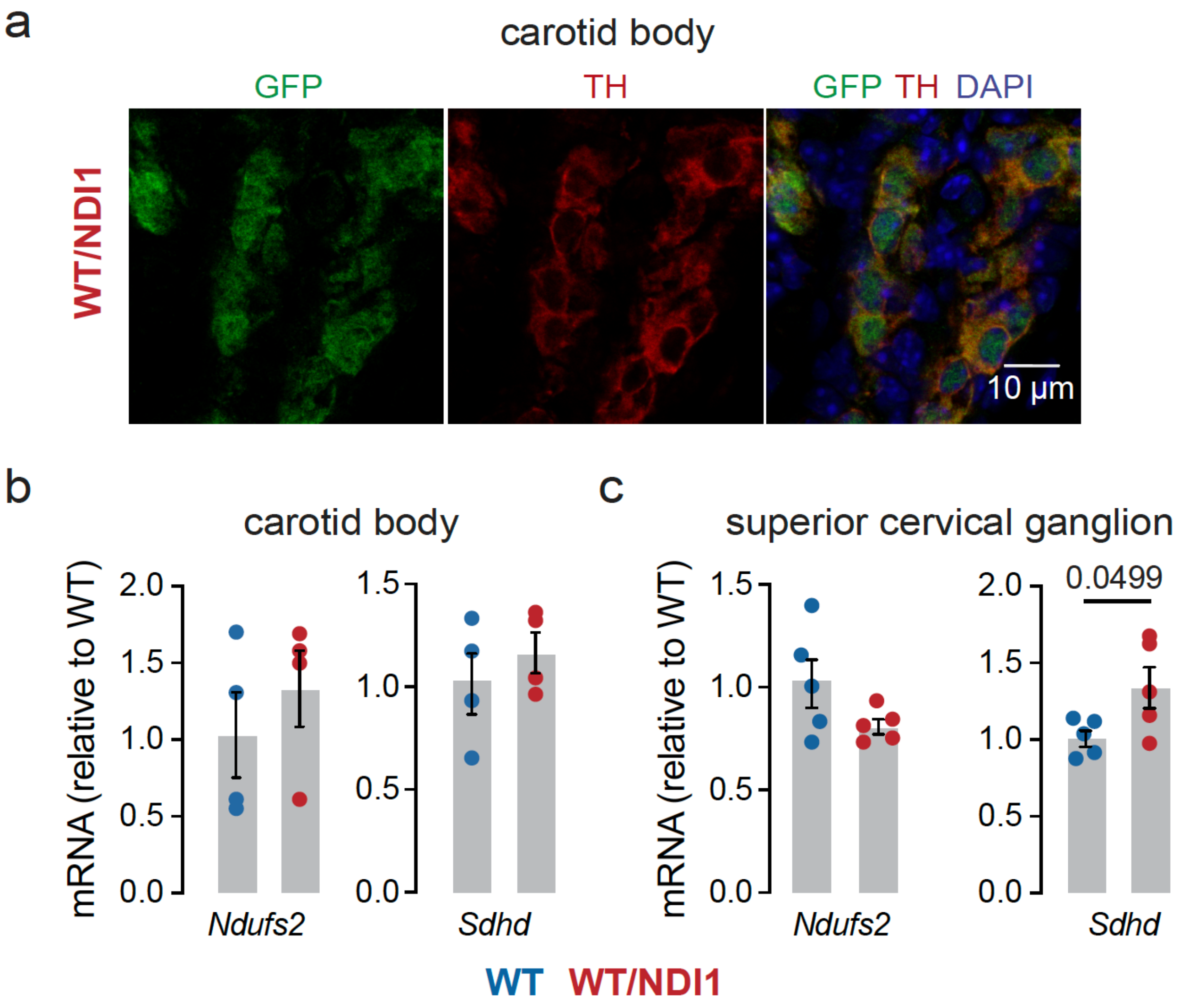

Supplementary Fig. 6. NDI1 and NDUFS2 expression in the carotid body of WT/NDI1 mice. a, Histological sections of the carotid body (CB) from WT/NDI1 mice illustrating colocalization of NDI1 (green fluorescent protein, GFP) and tyrosine hydroxylase (TH). DAPI was used to stain nuclei. Similar immunocytochemical studies were performed in n=4 mice. Calibration bar (10 μM) applies to all panels. **b, c,** Levels of *Ndufs2* and *Sdhd* mRNA expression relative to WT, in carotid body (**b**), and superior cervical ganglion (**c**, SCG), samples. Data are expressed as mean±SEM relative to WT. CB WT mice (*Ndufs2:* 1±0.28, n=4 replicates/group; *Sdhd*: 1±0.15, n=4 replicates/group), CB WT/NDI1 (*Ndufs2*: 1.3±0.25, n=4 replicates/group; *Sdhd:* 1.2±0.1, n=4 replicates/group). SCG WT mice (*Ndufs2:* 1±0.12, n=5 replicates/group; *Sdhd:* 1±0.05, n=5 replicates/group). SCG WT/NDI1 mice (*Ndufs2:* 0.8±0.04, n=5 replicates/group; *Sdhd*: 1.3±0.13, n=5 replicates/group). Statistically

significant P-values calculated by two-tailed unpaired t test are indicated. Source data are provided in a Source Data file.

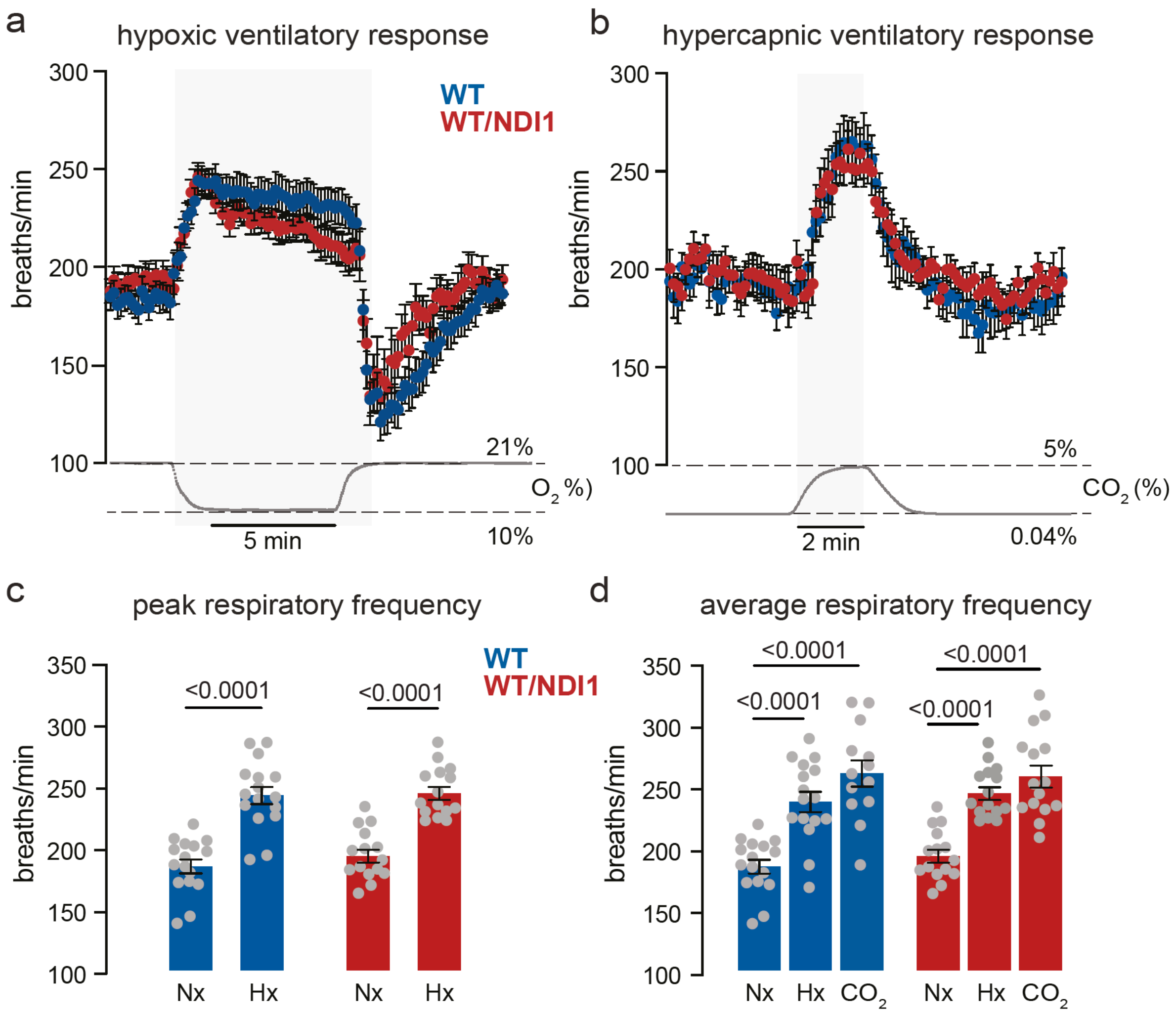

Supplementary Fig. 7. Acute hypoxic and hypercapnic ventilatory responses in WT/NDI1 mice. **a, b,** Time course of the increase in respiratory frequency (breaths/min) induced by hypoxia (**a,** Hx, 10% O_2), and hypercapnia (**b,** 5% CO_2), in WT mice (blue, Hx, n=16, CO_2, n=13) and WT/NDI1 mice (red, Hx, n=15, CO_2, n=15). Each data point is represented as mean±SEM. Changes in %O_2 and %CO_2 with time are represented at the bottom of each panel. **c,** Peak respiratory frequency (breaths/minute) recorded in normoxia (Nx, 21% O_2 tension) and during hypoxia (Hx, 10% O_2 tension) measured by plethysmography (see Methods). Individual data points are represented as grey dots. Mean±SEM values are: WT mice (Nx: 184±6; Hx: 241±7), WT/NDI1 mice (Nx: 192±5; Hx: 243±5). Number of mice as in panel **a.** Statistically significant P values calculated by two-tailed paired t test are indicated. **d,** Average respiratory frequency (breaths/minute) recorded in normoxia (Nx), during the first 80 s after reaching 10% O_2 in the chamber (Hx), and during hypercapnia (CO_2) (see Methods). Mean±SEM values are (values in normoxia are the same as in panel **c**): WT mice (Hx: 236±8; CO_2: 259±11), WT/NDI1 mice (Hx: 243±5; CO_2: 257±9). Number of mice as in panel **a** and **b** Statistically

significant P values calculated by one-way ANOVA followed by Dunnett's multiple comparisons test are indicated. Source data are provided in a Source Data file.

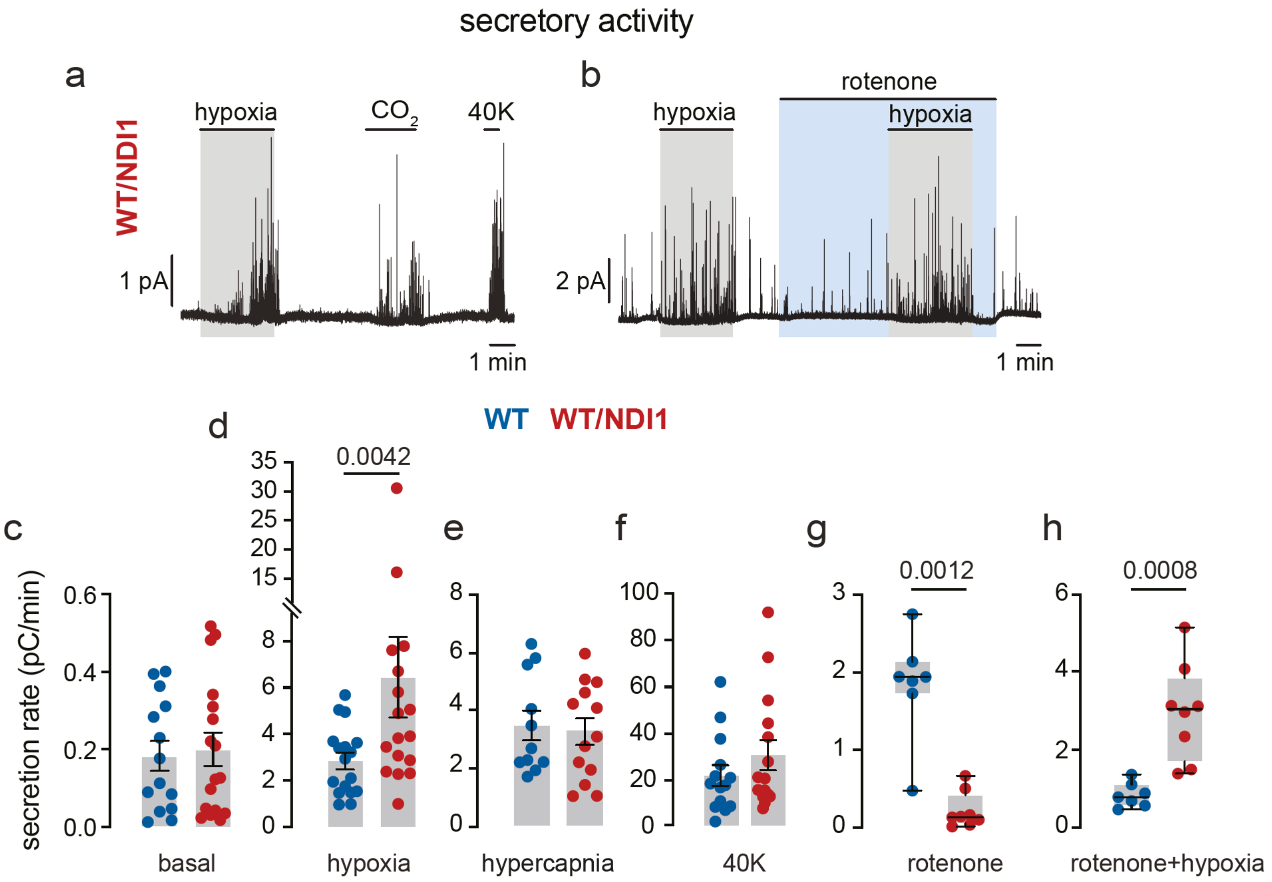

Supplementary Fig. 8. Secretory responses to hypoxia in glomus cells from WT/NDI1 mice. a, Secretory responses of glomus cells in carotid body (CB) slices from WT/NDI1 mice induced by hypoxia (O_2 tension ~15 mmHg), hypercapnia (20% CO_2) and depolarization with high potassium (40 mM K). **b,** Secretory responses of glomus cells in CB slices from WT/NDI1 mice to hypoxia and rotenone (5 μM). Note that rotenone did not occlude responsiveness to hypoxia. **c-f,** Average secretion rate (in picoCoulombs/min) in glomus cells during normoxia (basal, **c**) and during exposure to hypoxia (**d**), hypercapnia (**e**), and high K (**f**). Data are expressed as mean±SEM with data points superimposed. Normoxia (basal): WT mice (blue dots; 0.17±0.03, n=21/17 cell/ mice); WT/NDI1 mice (red dots; normoxia: 0.20±0.04, n=17/9 cells/mice). Hypoxia: WT mice (2.7±0.32, n=20/17 cells/mice); WT/NDI1 mice (6.5±1.7, n =17/9 cells/mice). Hypercapnia: WT mice (3.6±0.51, n=11/9 cells/mice); WT/NDI1 mice (3.4±0.46, n=13/9 cells/mice). 40K: WT mice (20.8±4.5, n=14/7 cells/mice): WT/NDI1 mice (29.5±6.5, n=15/9 cells/mice). **g, h,** Distribution of secretion rate (in picoCoulombs/min) induced by rotenone (**g**) and hypoxia in the presence of rotenone (5 μM) (**h**) in glomus cells in CB slices from WT mice (blue dots, rotenone: n=7/7 cells/mice; rotenone+hypoxia: n=7/7 cells/mice) and WT/NDI1 mice (red dots, rotenone: n=8/5 cells/mice; rotenone+hypoxia: n=8/5 cells/mice). The box plots represent median (middle line), 25th, 75th percentile (box), and largest and smallest values range

(whiskers). P values calculated with two-tailed Mann–Whitney test (**g**) and two-tailed unpair t test (**h**). Source data are provided in a Source Data file.

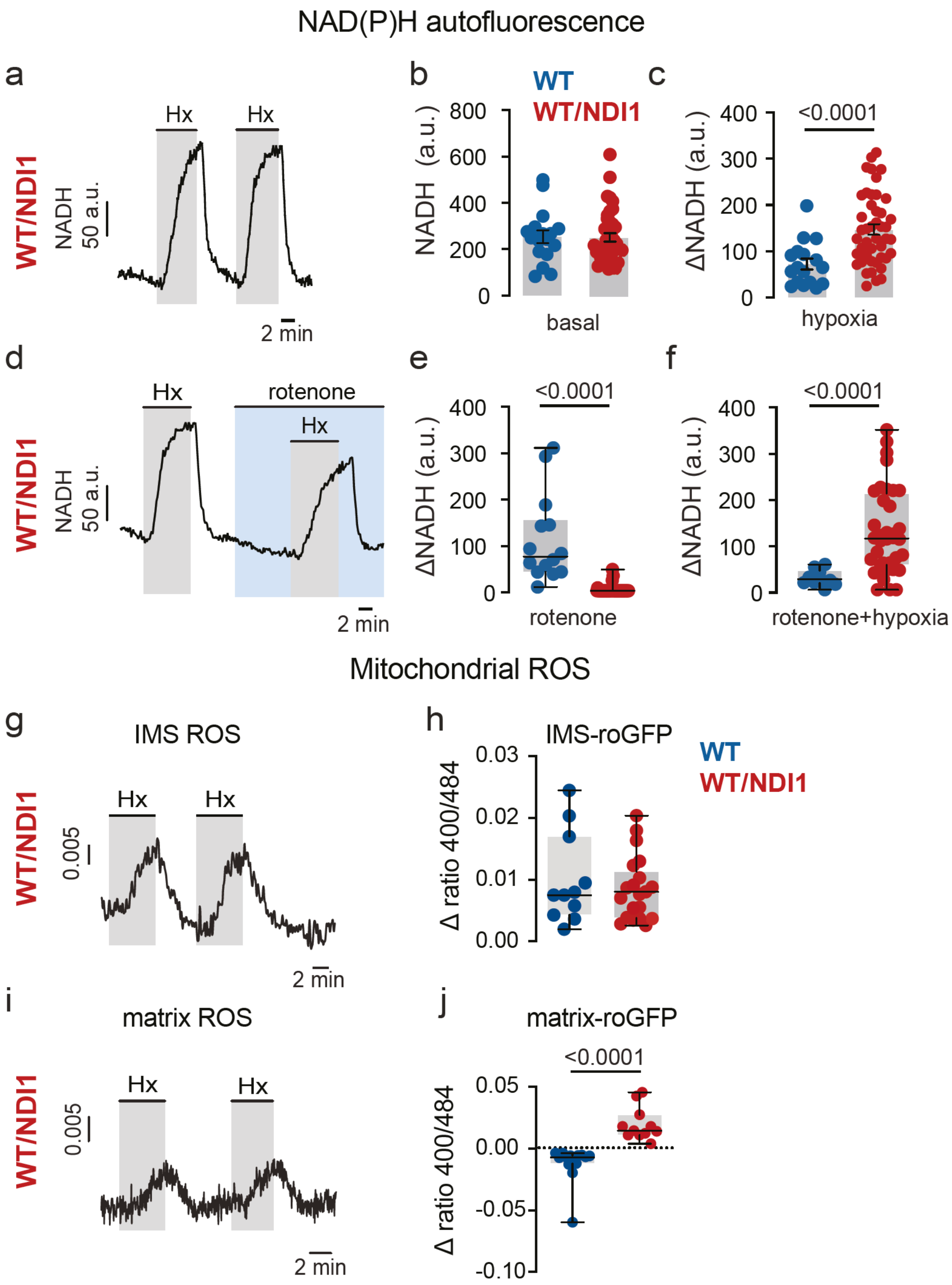
NAD(P)H autofluorescence
a
WT/NDI1
NADH
50 a.u.
Hx
Hx
2 min
b
WT
WT/NDI1
NADH (a.u.)
800
600
400
200
0
basal
c
ΔNADH (a.u.)
400
300
200
100
0
<0.0001
hypoxia
d
WT/NDI1
NADH
50 a.u.
Hx
rotenone
Hx
2 min
e
ΔNADH (a.u.)
400
300
200
100
0
<0.0001
rotenone
f
ΔNADH (a.u.)
400
300
200
100
0
<0.0001
rotenone+hypoxia
Mitochondrial ROS
g
IMS ROS
WT/NDI1
0.005
Hx
Hx
2 min
h
IMS-roGFP
Δ ratio 400/484
0.03
0.02
0.01
0.00
WT
WT/NDI1
i
matrix ROS
WT/NDI1
0.005
Hx
Hx
2 min
j
matrix-roGFP
Δ ratio 400/484
<0.0001
0.05
0.00
-0.05
-0.10

Supplementary Fig. 9. Mitochondrial signaling of hypoxia in glomus cells from WT/NDI1 mice. **a, d,** Changes in NAD(P)H autofluorescence recorded in dispersed carotid body (CB) glomus cells from WT/NDI1 mice in response to hypoxia (**a,** Hx, O_2 tension, ~15 mm Hg), rotenone (1 μM) and hypoxia in the presence of rotenone (**d**). **b,** Basal levels of NAD(P)H autofluorescence (arbitrary units, a.u.) in normoxia (O_2 tension, ~150 mmHg) in dispersed glomus cells from WT mice (blue dots, 254±28, n=17/6, cells/mice) and WT/NDI1 mice (red dots, 251±18, n=40/7 cells/mice). **c,** Increase in NAD(P)H autofluorescence (a.u.) in isolated glomus cells in response to hypoxia. WT mice (72±12, n=17/6 cells/mice); WT/NDI1 mice (147±11, n=45/7 cells/mice). In **b,** and **c,** data are mean±SEM. Statistically significant P-values calculated by two-tailed unpaired t test are represented. **e, f,** Box plots representing the increase in NAD(P)H autofluorescence in glomus cells in response to rotenone (**e**)**,** and rotenone plus hypoxia (**f**)**.** The box plots indicate median (middle line), 25th, 75th percentile (box), and largest and smallest values range (whiskers). Individual data points are superimposed. WT (rotenone: n=14/4 cells/mice; rotenone + hypoxia: n=10/4 cells/mice), and WT/NDI1 (rotenone: n=40/7 cells/mice; rotenone+hypoxia: n=36/7 cells/mice). Statistically significant P values calculated with two-tailed Mann–Whitney test are represented. **g, i,** Acute changes in reactive oxygen species at the mitochondrial intermembrane space (IMS ROS) (**g**)**,** or mitochondrial matrix (matrix ROS) (**i**)**,** of CB glomus cells from WT/NDI1 mice induced by hypoxia (Hx). **h, j**, Box plots representing hypoxia-induced increases (400/480 ratio) in IMS ROS (**h**) and matrix ROS (**j**)**,** in glomus cells in response to hypoxia. WT mice (IMS ROS: n=11/6 cells/mice; matrix ROS: n=15/8 cells/mice); WT/NDI1 mice (IMS ROS n=21/6 cells/mice; matrix ROS: n=11/4 cells/mice). The boxplots indicate median (middle line), 25th, 75th percentile (box), and largest and smallest values range (whiskers). Individual data points are superimposed. P values were calculated with two-tailed Mann–Whitney tests. Source data are provided in a Source Data file.

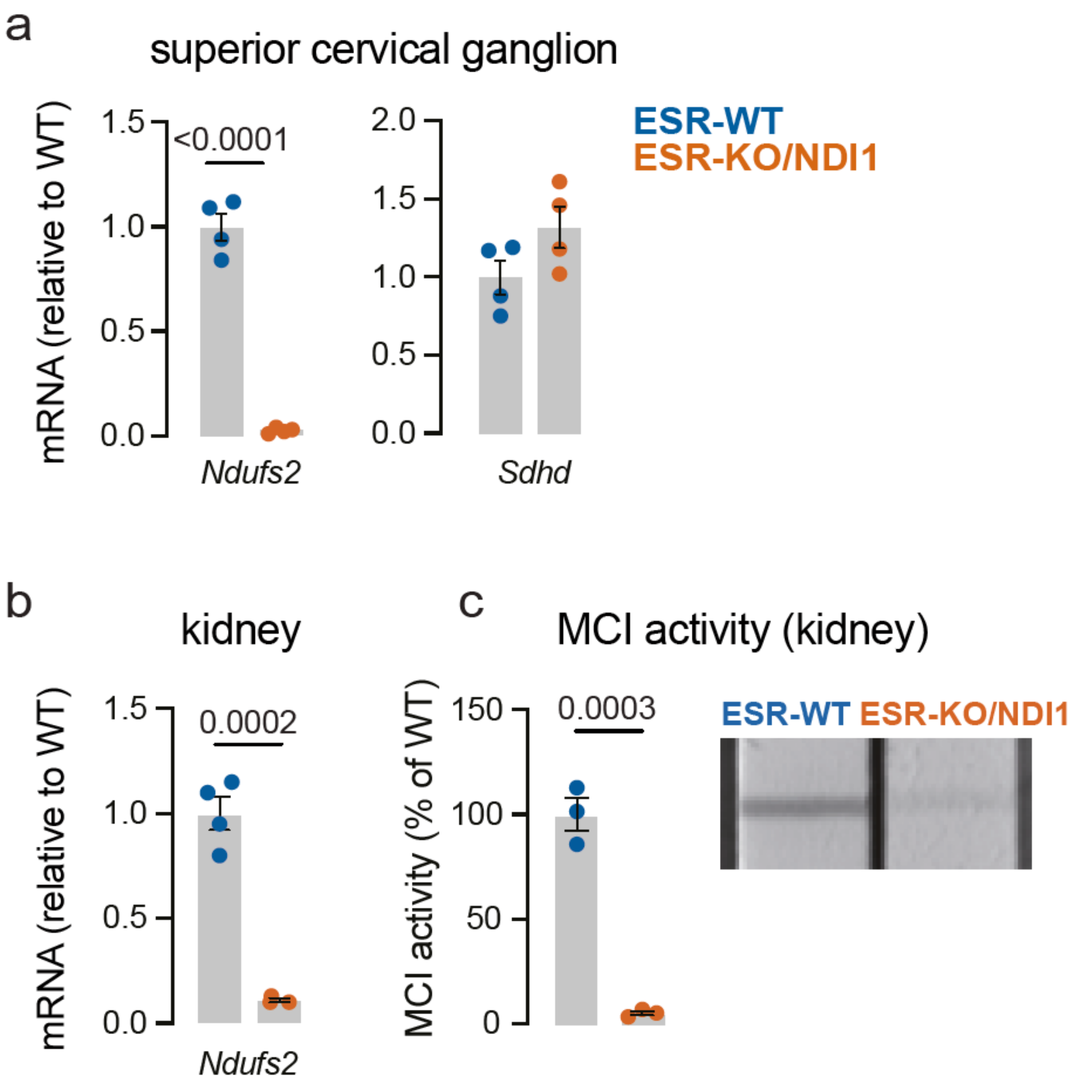

Supplementary Fig. 10. NDUFS2 expression and MCI activity in adult conditional genetically modified mice. a, mRNA levels of *Ndufs2* (left) and *Sdhd* (right) relative to WT, in superior cervical ganglion samples from ESR-WT mice (*Ndufs2*: 1±0.07; n=4/group, *Sdhd*: 1±0.11, n=4 replicates/group), ESR-KO/NDI1 mice (*Ndufs2*: 0.025±0.006, n=4 replicates/group, *Sdhd*: 1.32±0.13, n=4 replicates/group). **b,** mRNA levels of *Ndufs2* relative to WT, in kidney samples from ESR-WT mice (*Ndufs2*: 1±0.08, n=4 replicates/group) and ESR-KO/NDI1 mice (*Ndufs2*: 0.11±0.01, n=3 replicates/group). **c,** Measurement of MCI activity in kidney cells from ESR-WT and ESR-KO/NDI1 mice using a dipstick assay. **c,** Levels of kidney MCI activity (in % of WT) in kidney cells from ESR-WT mice (100±7.8, n=3 replicates/group) and ESR-KO/NDI1 mice (5.1±1, n=3 replicates/group). Data in **a**, **b** and **c** are expressed as mean±SEM. Statistically significant P-values calculated by two-tailed unpaired t test are indicated. Source data are provided in a Source Data file.

NADH autofluorescence in single glomus cells

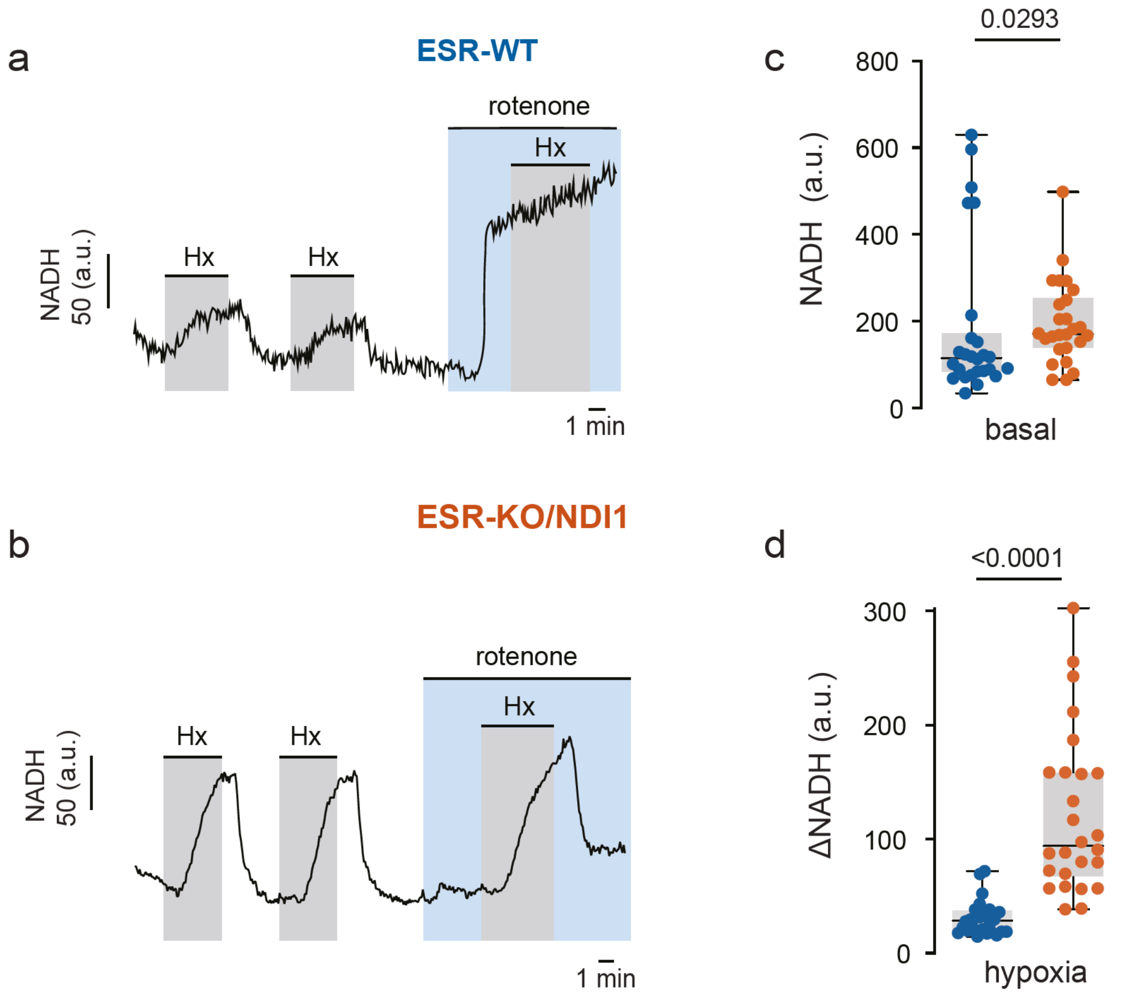

Supplementary Fig. 11. Mitochondrial responses to hypoxia in MCI-deficient mice with conditional transgenic NDI1 expression in adulthood. a, b, Representative recordings of changes in NADH autofluorescence (in arbitrary units, a.u.) elicited by hypoxia (Hx), rotenone (1 μM), and hypoxia plus rotenone in glomus cells from ESR-WT (**a**) and ESR-KO/NDI1 (**b**) mice. **c, d,** Box plots representing basal level of NADH (**c**) and the increase in NADH autofluorescence in response to hypoxia (**d**)**,** recorded in dispersed glomus cells from ESR-WT (blue dots n=26/5 cells/mice) and ESR-KO/NDI1 (brown dots n=26/7 cells/mice) mice. The box plots indicate median (middle line), 25th, 75th percentile (box), and largest and smallest values range (whiskers). Represented P values were calculated with two-tailed Mann–Whitney test. Mean±SEM values were: basal (ESR-WT mice: 187±35; ESR-KO/NDI1 mice: 196±19), increase in response to hypoxia (ESR-WT mice: 31±3; ESR-KO/NDI1 mice: 121±14). Source data are provided in a Source Data file.
