# Supplementary material for: Transgenic NADH dehydrogenase restores oxygen regulation of breathing in mitochondrial complex I-deficient mice
Source: Nat Commun. 2023 Mar 1;14:1172. doi: 10.1038/s41467-023-36894-2 (PMC9977773; doi:10.1038/s41467-023-36894-2)
Supplement: Supplementary file 3 — Reporting Summary [file 41467_2023_36894_MOESM3_ESM.pdf]

nature portfolio

Corresponding author(s): Lopez-Barneo J

Last updated by author(s): 2023/02/09

Reporting Summary

Nature Portfolio wishes to improve the reproducibility of the work that we publish. This form provides structure for consistency and transparency in reporting. For further information on Nature Portfolio policies, see our Editorial Policies and the Editorial Policy Checklist.

Statistics

For all statistical analyses, confirm that the following items are present in the figure legend, table legend, main text, or Methods section.

n/a	Confirmed	
[ ]	[x]	The exact sample size (*n*) for each experimental group/condition, given as a discrete number and unit of measurement
[ ]	[x]	A statement on whether measurements were taken from distinct samples or whether the same sample was measured repeatedly
[ ]	[x]	The statistical test(s) used AND whether they are one- or two-sided<br>*Only common tests should be described solely by name; describe more complex techniques in the Methods section.*
[x]	[ ]	A description of all covariates tested
[ ]	[x]	A description of any assumptions or corrections, such as tests of normality and adjustment for multiple comparisons
[ ]	[x]	A full description of the statistical parameters including central tendency (e.g. means) or other basic estimates (e.g. regression coefficient) AND variation (e.g. standard deviation) or associated estimates of uncertainty (e.g. confidence intervals)
[ ]	[x]	For null hypothesis testing, the test statistic (e.g. *F*, *t*, *r*) with confidence intervals, effect sizes, degrees of freedom and *P* value noted<br>*Give P values as exact values whenever suitable.*
[x]	[ ]	For Bayesian analysis, information on the choice of priors and Markov chain Monte Carlo settings
[x]	[ ]	For hierarchical and complex designs, identification of the appropriate level for tests and full reporting of outcomes
[ ]	[x]	Estimates of effect sizes (e.g. Cohen's *d*, Pearson's *r*), indicating how they were calculated

Our web collection on statistics for biologists contains articles on many of the points above.

Software and code

Policy information about availability of computer code

Data collection	Pulse v8.80, 12-Dec-2006 (HEKA Elektronik)<br>Aquacosmos 2.6 (Hamamatsu)<br>Leica Application Suite X (Leica Microsystems)<br>LabVIEW 12.0 (Scientifica)<br>IOX2 software (EMKA Technologies)<br>Live Acquisition software (T.I.L.L Photonics)<br>ImageQuant LAS 4000mini, GE Healthcare
Data analysis	Prism Version 8.2.1 (279) for MacOS<br>Igor Pro 4.08 carbon<br>ImageQuant TL software (GE Healthcare)<br>Fiji 2.0.0

For manuscripts utilizing custom algorithms or software that are central to the research but not yet described in published literature, software must be made available to editors and reviewers. We strongly encourage code deposition in a community repository (e.g. GitHub). See the Nature Portfolio guidelines for submitting code & software for further information.

Data

Policy information about availability of data

All manuscripts must include a data availability statement. This statement should provide the following information, where applicable:

- Accession codes, unique identifiers, or web links for publicly available datasets
- A description of any restrictions on data availability
- For clinical datasets or third party data, please ensure that the statement adheres to our policy

A data availability statement has been included in the text:
The data that support the findings of this study are available from the corresponding author upon reasonable request.
Authors can confirm that all relevant data are included in the paper and/or its supplementary information/extended data files. Source data are provided as a Source Data file.

Human research participants

Policy information about studies involving human research participants and Sex and Gender in Research.

Reporting on sex and gender	*Use the terms sex (biological attribute) and gender (shaped by social and cultural circumstances) carefully in order to avoid confusing both terms. Indicate if findings apply to only one sex or gender; describe whether sex and gender were considered in study design whether sex and/or gender was determined based on self-reporting or assigned and methods used. Provide in the source data disaggregated sex and gender data where this information has been collected, and consent has been obtained for sharing of individual-level data; provide overall numbers in this Reporting Summary. Please state if this information has not been collected. Report sex- and gender-based analyses where performed, justify reasons for lack of sex- and gender-based analysis.*
Population characteristics	*Describe the covariate-relevant population characteristics of the human research participants (e.g. age, genotypic information, past and current diagnosis and treatment categories). If you filled out the behavioural & social sciences study design questions and have nothing to add here, write "See above."*
Recruitment	*Describe how participants were recruited. Outline any potential self-selection bias or other biases that may be present and how these are likely to impact results.*
Ethics oversight	*Identify the organization(s) that approved the study protocol.*

Note that full information on the approval of the study protocol must also be provided in the manuscript.

Field-specific reporting

Please select the one below that is the best fit for your research. If you are not sure, read the appropriate sections before making your selection.

☒ Life sciences ☐ Behavioural & social sciences ☐ Ecological, evolutionary & environmental sciences

For a reference copy of the document with all sections, see nature.com/documents/nr-reporting-summary-flat.pdf

Life sciences study design

All studies must disclose on these points even when the disclosure is negative.

Sample size	We defined the number of independent measurements necessary to ascertain if two parameters have similar or different values based on the experimental evidence and the previous experience in our laboratory. See references 7 and 8 in the main text.
Data exclusions	No data were excluded.
Replication	The data obtained from in vivo and in vitro preparations were systematically and clearly replicated in several independent experiments performed in different days (animals or in vitro cultures) as stated in the figure legends
Randomization	Randomization and blind experiments have been performed to reduce bias in animal studies. For example, in the in vitro preparation of CB slices or dispersed cells, animals were randomized. Randomizing the different animal models used (WT, KO, WT/NDI1 and KO/NDI1) we ensure that the WT animals is not always the first one and the KO/NDI1 is not always the last one to be processed. The tissues of the last one processed might just be left in the Petri dish or in the enzyme for a few seconds longer which could already have a significant impact on cell/slice viability and, thus, could change the outcome of the experiment. In this way, randomizing animals could contribute to eliminate experimental bias and ensure higher confidence in experimental data.
Blinding	The animals are marked with numbers. In the functional experiments, both in vivo and in vitro, pairs of animals were used to facilitate comparison. The researcher only knows the number of the animal, without knowing the genotype, until the end of the analysis of the experimental data. For plethysmography, a technician places the animals in the cages and performs the plethysmography and then the

analysis is performed by the researcher, who does not know the genotype of the animals. Subsequently, the animals are re-genotyped to confirm the genotype and include the results in the corresponding group. In the in vitro experiments, the dissection of the tissues is performed by the technician and from there the experiments of amperometry, microfluorimetry, etc, are performed and analyzed blindly. Afterwards, the animals are re-genotyped to confirm the genotype and the results are included in the corresponding group.

Reporting for specific materials, systems and methods

We require information from authors about some types of materials, experimental systems and methods used in many studies. Here, indicate whether each material, system or method listed is relevant to your study. If you are not sure if a list item applies to your research, read the appropriate section before selecting a response.

Materials & experimental systems

n/a	Involved in the study	
☐	☒	Antibodies
☒	☐	Eukaryotic cell lines
☒	☐	Palaeontology and archaeology
☐	☒	Animals and other organisms
☒	☐	Clinical data
☒	☐	Dual use research of concern

Methods

n/a	Involved in the study	
☒	☐	ChIP-seq
☒	☐	Flow cytometry
☒	☐	MRI-based neuroimaging

Antibodies

Antibodies used

*Primary antibodies:
-TH (1:2500 dilution, NB300-109, Novus Biological Inc.)
-TH (1:200 dilution, AB1542, Millipore)
-NDUFS2 (1:200 dilution, ab192022,Abcam)
-GFP (1:400 dilution, 1010, Aves Labs Inc)
*Fluorescent secondary antibodies:
Alexa Fluor™ 488: Donkey anti-Sheep IgG (H+L) Cross-Adsorbed Secondary Antibody, Invitrogen (A11015). Dilution 1:500
Alexa Fluor™ 568: Goat anti-Rabbit IgG (H+L) Cross-Adsorbed Secondary Antibody, Invitrogen (A11011). Dilution 1:500
*Nuclei were labeled with 4′,6′-diamidino-2-phenylindole (DAPI)

Validation

Mouse TH primary antibody has previously been used in our laboratory (see Gao et al., J Physiol 2017 in reference list).
NDUFS2 primary antibody (1:200 dilution, ab192022,Abcam) has been used by other investigators (see, for example, Gregg T et al. Obesity-dependent CDK1 signaling stimulates mitochondrial respiration at complex I in pancreatic ß-cells. J Biol Chem 294:4656-4666 (2019).
-GFP (1:400 dilution, 1010, Aves Labs Inc) primary antibody (Anti-Green Fluorescent Protein Antibody - Aves Labs https://www.aveslabs.com)

Animals and other research organisms

Policy information about studies involving animals; ARRIVE guidelines recommended for reporting animal research, and Sex and Gender in Research

Laboratory animals

TH-NDUFS2 (B6/129SV background) Laboratory of José López-Barneo. (Fernandez Aguera et al., Cell Metab 2015 in reference list)
LSL-GFP-NDI1 and LSL-NDI1(C57BL/6) Laboratory of Navdeep S. Chandel (Mac Elroy et al., Cell Metab 2020, in reference list)
TH-NDUFS2-LSL-NDI1 and TH-NDUFS2-LSL-GFP-NDI1 were generated in our animal facility (Instituto de Biomedicina de Sevilla)
ESR-NDUFS2 (B6/129SV background) Laboratory of José López-Barneo. (Fernandez Aguera et al., Cell Metab 2015 in reference list)
ESR-NDUFS2-LSL-NDI1 and ESR-NDUFS2-LSL-GFP-NDI1 were generated in our animal facility (Instituto de Biomedicina de Sevilla).
For experiments performed on embryonic knockout mouse models we always used >2 months old mice of both sexes.
Adult wildtype and conditional knockout mice ≥2 months (2-14 months) old were fed with a tamoxifen-containing diet for a month followed by normal diet until they were used for experiments (20-270 days later).

Wild animals

No wild animals were used in this study.

Reporting on sex

For in vivo experiments, the sex of the animals was always noted, but males and females were both used in the study. In most experiments the number of males and females used was similar. Animals were separated by sex when we studied parameters (e.g. animal weigth) that are notably affected by gender

Field-collected samples	The study did not involve samples collected from the field
Ethics oversight	All procedures were approved by the Institutional Committee of the University of Seville for Animal Care and Use (2012PI/LB02 and 22-09-15-332). Handling of the animals was conducted in accordance with the European Community Council directives 86/609/EEC, and 2010/63/EU for the Care and Use of Laboratory Animals.

Note that full information on the approval of the study protocol must also be provided in the manuscript.
